# Development and validation of monoclonal antibodies against N6-methyladenosine for the detection of RNA modifications

**DOI:** 10.1371/journal.pone.0223197

**Published:** 2019-10-02

**Authors:** Shun Matsuzawa, Yuka Wakata, Fumiya Ebi, Masaharu Isobe, Nobuyuki Kurosawa

**Affiliations:** 1 Graduate School of Innovative Life Science, University of Toyama, Toyama-shi, Toyama, Japan; 2 Medical & Biological Laboratories Co., Ltd., Akaho, Komagane, Nagano, Japan; 3 Graduate School of Science and Engineering for Education, University of Toyama, Gofuku, Toyama-shi, Toyama, Japan; 4 Laboratory of Molecular and Cellular Biology, Faculty of Science and Engineering, Graduate School, University of Toyama, Gofuku, Toyama-shi, Toyama, Japan; University of Toronto, CANADA

## Abstract

RNA contains various chemical modifications, among which N6-methyladenosine (m6A) is the most prevalent modified nucleotide in eukaryotic mRNA. Emerging evidence suggests that m6A plays an important role in regulating a variety of cellular functions by controlling mRNA processing, translation and degradation. Because m6A is not detectable by standard chemical modification-based approaches, immunological methods, such as ELISA, immunoblotting, immunohistochemistry, m6A RNA immunoprecipitation sequencing and m6A individual-nucleotide resolution cross-linking and immunoprecipitation, have been employed to detect m6A in RNA. Although the most important factor determining the success of these methods is the integrity of highly specific antibodies against m6A, the development of m6A-specific monoclonal antibodies has been challenging. We developed anti-m6A monoclonal antibodies using our recently developed single cell-based monoclonal antibody production system. The binding of one selected antibody, #B1-3, to RNA oligoribonucleotide containing a single m6A had an equilibrium dissociation constant of 6.5 nM, and this antibody exhibited negligible binding to oligoribonucleotides containing a single N1-methyladenosine and unmodified adenosine. The binding was competed by the addition of increasing concentrations of N6-methyl-ATP but not N1-methyl-ATP or ATP. Furthermore, this mAb specifically crosslinked m6A-containing oligoribonucleotide by ultraviolet light, resulting in the induction of cDNA truncation at m6A position. These results show the feasibility of using the validated m6A monoclonal antibody for the specific detection of m6A in RNA.

## Introduction

RNAs contain over 100 distinct posttranscriptional nucleotide modifications, of which N6-methyladenosine (m6A) is the most abundant internal modification in eukaryotic messenger RNA (mRNA) [1,2] [3–6]. In addition to internal m6A, N6,2'-O-dimethyladenosine (m6Am) is exclusively found at the +1 position in the 5' cap in mRNA [7,8]. Although a reliable method for the genome-wide analysis of C5-methyl-cytosine patterns in DNA has been established by the selective chemical deamination of cytosine to uracil with bisulfite treatment, there is no chemical method that results in the selective modification and detection of m6A in RNA [9]. Thus, the precise location, temporal dynamics and functional importance of m6A in RNA remain incompletely understood. Recently, RNA immunoprecipitation by an anti-m6A antibody followed by high-throughput sequencing of the captured RNA fragments (m6A-Seq) enabled mapping of the locations of m6A at ~100 nt resolution. To map the positions of m6A in RNAs more precisely, m6A individual-nucleotide resolution cross-linking and immunoprecipitation (miCLIP) has been developed, revealing that m6A is mainly enriched in intragenic regions, 3′ untranslated regions and near stop codons in mRNAs with a consensus sequence of RRACH (R corresponds to G or A; H corresponds to A, C or U) [3,10]. The topology of m6A in RNA revealed by m6A-seq and miCLIP provides evidence that m6A regulates RNA metabolism, including splicing, nuclear export, translation and stability [11–15]. Currently, several polyclonal and monoclonal antibodies against m6A have been used. However, the polyclonal antibody comprises a mixed population of antibodies with activity that vary from batch to batch, which hampers the reproducibility and comparability of the results. Monoclonal antibody (mAb) is reproducible, recognize a defined epitope with a given affinity and specificity, and therefore produce more consistent results. However, attempts to produce m6A-specific mAb by traditional methods have been challenging. Because m6A is a non-immunogenic small molecule with subtle structural differences in the nucleobase [3,15–18].

We recently reported a method for the high-throughput production of phosphorylation site-specific mAbs by developing a fluorescence-activated cell sorting (FACS)-based antigen-specific plasma cell (PC) isolation method termed FIXAA, in which antigen-specific PCs can be isolated by the intracellular labeling of paraformaldehyde-fixed lymph node cells with a fluorescently labeled antigen combined with an antibody against the immunoglobulin [19]. By using this technology, we achieved the efficient production of anti-m6A mAbs from guinea pigs immunized with oligoribonucleotide containing a single m6A (m6A-oligo). Surface plasmon resonance (SPR) analysis revealed that one selected antibody clone, #B1-3, specifically bound to m6A-oligo, but not to oligoribonucleotide containing a single m1A (m1A-oligo) or unmodified adenosine (A-oligo). The m6A binding was competed by the addition of N6-methyl-ATP but not N1-methyl-ATP or ATP. Furthermore, this mAb was crosslinked to m6A residues in RNA by UV light, resulting cDNA truncation at the m6A position. These data show the high selectivity of this mAb for m6A and demonstrate its ability to detect m6A in RNA.

## Materials and methods

### Materials

Rabbit polyclonal antibodies against m6A (SYSY and RN131P) were purchased from Synaptic Systems (https://www.sysy.com) and MBL (http://ruo.mbl.co.jp), respectively. Mouse monoclonal antibody against m6A (17-3-4-1) was purchased from Enzo Life Sciences (http://www.enzolifesciences.com). DyLight-labeled streptavidin and goat secondary antibodies against guinea pig IgG were purchased from Abcam (http://www.abcam.co.jp). Protein A and oligo(dT)25 Dynabeads were purchased from Thermo Fisher Scientific (http://www.thermoscientific.com). T7 RNA polymerase, nucleotides and poly(A)-enriched RNA were purchased from Takara Bio (https://www.takarabio.com). N1-methyladenosine-5'-triphosphate (m1ATP) and N6-methyladenosine-5'-triphosphate (m6ATP) were obtained from TriLink BioTechnologies (https://www.trilinkbiotech.com). Biotinylated and unbiotinylated oligoribonucleotides containing a single m6A (m6A-oligo), m1A (m1A-oligo) or unmodified adenosine (A-oligo) were purchased from Integrated DNA Technologies (https://sg.idtdna.com). A 34 nt Snord2 oligoribonucleotide containing a single m6A was synthesized by TsukuBio (http://www.tos-bio.com). The oligoribonucleotide sequences are shown in S1 Table and S2 Table. DNA fragments containing eleven Dam methylation sites were synthesized by Eurofins and subcloned into pEX-A2J1 (https://www.eurofinsgenomics.jp). The nucleotide sequence is shown in S1 File. IRDye800CW donkey anti-guinea pig IgG (H + L) and IRDye680LT donkey anti-mouse IgG (H + L) were purchased from LI-COR Biosciences (http://www.licor.com/). Female Hartley guinea pigs were purchased from Japan SLC, Inc. (http://jslc.co.jp).

### Immunization

All animal studies were approved by the Committee for Laboratory Animal Care and Use at University of Toyama, and the experiments were carried out in accordance with approved guidelines (Protocol Number: A2016ENG-3). m6A-oligo were crosslinked with keyhole limpet hemocyanin (KLH) via sodium periodate as previously described [20]. Briefly, 3.8 mg of m6A-oligo was oxidized with 0.1M of sodium periodate at room temperature for 20 min. Excess periodate was then quenched with 1M of ethylene glycol. After five minutes of incubation at room temperature, the reaction product was added in 5% potassium carbonate containing 11.2 mg of KLH and incubated at room temperature for 1 h. After the addition of NaBH_4_ followed by over night incubation at room temperature, the KHL-conjugated m6A-oligo was purified and concentrated with Centricut (Kurobo, https://www.kurabo.co.jp) by repeated washing with PBS. Guinea pigs were immunized three times intramuscularly at the tail base with 200 μl of a 50:50 water-in-oil TiterMax Gold adjuvant emulsion containing the m6A-oligo conjugated to KLH. At 1 week after the final immunization, the iliac lymph nodes were surgically removed from two individual female Hartley guinea pigs under euthanasia with a barbiturate overdose.

### Isolation of m6A-specific plasma cells by FIXAA

Biotinylated m6A-oligo (1.6 μM) and A-oligo (1.6 μM) were conjugated to DyLight streptavidin 488 (0.4 μM) and DyLight streptavidin 550 (0.4 μM) to generate fluorescently labeled m6A-oligo (m6A-oligo-488) and A-oligo (A-oligo-550), respectively. An aliquot of 25 μl of each solution was combined in 200 μl of phosphate-buffered saline with 0.1% Triton X-100 (PBST) containing 10 μM biotin, DyLight 650-conjugated goat anti-guinea pig IgG (IgG-650) and 400 units of RNaseOUT (Thermo Fisher Scientific) and used as an intracellular staining solution. FIXAA was performed as described previously [19]. Briefly, iliac lymph node cells were fixed in 2% paraformaldehyde-PBS for 10 min on ice and resuspended in 250 μl of intracellular staining solution. After incubation for 10 min on ice, the cells were then diluted with 3 ml of ice-cold PBS containing 1 μM DAPI and analyzed by FACS. Single-cell sorting and data analysis were performed using a JSAN flow cytometer equipped with an automatic cell deposition unit (http://baybio.co.jp). The channels used were as follows: IgG-650 was in the FL-5 channel, m6A-oligo-488 was in the FL-1 channel, A-oligo-550 was in the FL-2 channel, and DAPI was in the FL-7 channel.

### Molecular cloning of immunoglobulin variable genes from single cells and recombinant antibody production

Immunoglobulin variable heavy (V_H_) and light chain (V_L_) genes were amplified from single cells by 5′-rapid amplification of cDNA end (5’-RACE) PCR as previously described [20]. The PCR-amplified V gene fragments were joined to their respective DNA cassettes to build linear immunoglobulin heavy (IgH) and light (IgL) genes by TS-jPCR or subcloned into a mammalian expression vector by TS homologous recombination to generate IgH and IgL expression plasmids [21,22]. The cognate pairs of linear IgH and IgL genes were cotransfected into 293FT cells grown in 96-well culture dishes with FuGENE HD Transfection Reagent (Promega, https://www.promega.com) and used for in-cell ELISA screening. The cognate pairs of IgH and IgL expression plasmids were cotransfected into Expi293 cells according to the manufacturer’s protocol, and the antibodies expressed in the cell culture media were purified with protein A column chromatography.

### Surface plasmon resonance (SPR) analysis

Biotinylated RNA oligonucleotides were immobilized on streptavidin SA sensor chips (GE Healthcare, https://www.gelifesciences.com). Briefly, two flowcells were prepared, and one of these flowcells served as a negative control (biotin), while biotinylated RNA oligonucleotide was injected into the other to obtain an immobilization level of 20 response units (RU) at a flow rate of 10 μl/min. The interaction assays involved injections of 5 different dilutions of anti-m6A antibodies (0, 1, 3, 9, 27 nM), which were flowed over the RNA oligonucleotide and negative control surfaces, followed by a 3 min washing step with HBS-N buffer to induce the dissociation of the complexes formed. All SPR experiments were performed with a Biacor T100 using HBS-EP+ buffer at a flow rate of 30 μl/min. At the end of each cycle, the sensor chip surface was regenerated by the injection of 0.1 M citric acid, pH 3. The sensorgrams corresponding to the RNA oligonucleotide signal were subtracted from the negative control. The single cycle kinetic curves were fitted on the basis of 1:1 binding to estimate the association and dissociation rate constants.

### In-cell ELISA

293FT cells transfected with cognate pairs of linear IgH and IgL genes were fixed in paraformaldehyde, permeabilized with PBST and stained with intracellular staining solution containing m6A-oligo-488, A-oligo-550 and IgG-650. The images were captured with an Operetta High Content Imaging System and were analyzed with a Columbus Image Data Storage and Analysis System (PerkinElmer, http://www.perkinelmer.com).

### ELISA

Biotinylated m6A-, m1A- or A-oligo (5 pmol) was immobilized on streptavidin-coated 96-well plates (Sumitomo Bakelite Co., Ltd.) in 50 μl PBS at 4°C followed by washing with PBS. Competition ELISA was performed with purified mAb (3 ng) mixed with a series of competitors in 50 μl PBS. The mixture was added to each well and incubated for 3 h at room temperature, followed by washing with PBS. The bound antibodies were detected by alkaline phosphatase (AP)-conjugated goat anti-guinea pig IgG (Abcam) with the BluePhos Microwell Substrate Kit (KPL, https://www.seracare.com) and quantified with a Tecan GENios microplate reader (TECAN, https://lifesciences.tecan.com).

### Dot blot screening of antibodies

Either 30 or 300 pM of m6A-, m1A- or A-oligo was spotted onto a NitroPure membrane (GVS, http://www.gvs.com). After UV crosslinking, the membranes were blocked for 1 h with Blocking One reagent (Nakarai Tesque, https://www.nacalai.co.jp) and incubated with purified antibodies (1 μg/mL) overnight at 4°C. After washing with PBST, the signals were visualized by IRDye800CW donkey anti-guinea pig IgG with an Odyssey imaging system (LI-COR).

### Immunoblotting

RNAs were synthesized using linearized plasmids with 0.8 kb of mouse microtubule-associated protein 2 cDNA as a template. The reaction was performed using T7 RNA polymerase in a reaction mix in which the ratio of m6ATP relative to ATP was 0 ~ 20%. The incorporation efficiency of m6ATP by T7 RNA polymerase has been reported to be almost the same level as that of ATP [23]. The transcripts were purified by phenol:chloroform extraction and ethanol precipitation. Immuno-Northern blotting was performed as follows: RNA was denatured by incubation at 65°C for 5 min, electrophoresed on a denaturating 1% agarose gel, blotted onto a NitroPure membrane under neutral conditions (20× SSC, pH 7.0) and UV crosslinked. Incubation with anti-m6A antibody (1 μg/mL) was performed for 12 h at 4°C in Block One blocking buffer. Subsequently, the blots were washed three times with PBST and incubated with secondary antibody (1:10,000, IRDye800CW donkey anti-guinea pig IgG) in blocking buffer for 1 h at room temperature. After washing with PBST, the membranes were visualized by the Odyssey imaging system (LI-COR). Immuno-Southern blotting was performed as follows: plasmid DNA extracted from the *dam*+ (Top10; Invitrogen) and *dam*^-^ *E*. *coli* strains (K12 ER2925; New England Biolabs) was loaded onto a 1% agarose gel, soaked in denaturing solution (1.5 M NaCl and 0.5 M NaOH) and neutralizing solution (1.5 M NaCl and 0.5 M Tris·HCl, pH 7.0) and then transferred onto a NitroPure membrane. The membrane was then UV crosslinked and blotted with anti-m6A antibody as described above. Gel images were acquired after ethidium bromide staining with the E-BOX Electrophoresis Gel Photodocumentation System (VILBER, https://www.vilber.com/e-box/).

### Immunohistochemical detection of m6A-containing RNA

Human fibroblasts grown on collagen-coated glass slides were fixed for 5 min with 4% paraformaldehyde-PBS and permeabilized with PBST. The cells were incubated with #B1-3 antibody and anti-tubulin mAb with or without competitor nucleotides and then stained with secondary antibodies corresponding to the primary antibodies. Human fibroblasts fixed with 70% ethanol were treated with or without ribonuclease A (RNase A) at 37°C for overnight and then stained with #B1-3 and propidium iodide (PI). The images were captured using a Leica TCS SP8 confocal laser scanning microscope (https://www.leica-microsystems.com).

### m6A individual-nucleotide resolution crosslinking and immunoprecipitation (miCLIP)

Each 4 pmol of biotinylated m6A-oligo, m1A-oligo or A-oligo was incubated with 2 μg of #B1-3 or control IgG in 20 μl of IP buffer (50 mM Tris pH 7.4, 100 mM NaCl and 0.05% NP40) for 30 min at 4°C. The solution was then crosslinked with 0.2 J cm^−2^ UV light (254 nm). The antibody-oligoribonucleotide complex was precipitated with protein A Dynabeads (Thermo Scientific) and then washed three times with IP buffer. After immunoprecipitation, the antibody-bound biotinylated oligoribonucleotides were quantified with alkaline phosphatase-conjugated streptavidin (VECTOR Laboratories, https://vectorlabs.com). The antibody-oligoribonucleotide complex was subject to SDS-PAGE, blotted on a nitrocellulose membrane and then probed with alkaline phosphatase-conjugated streptavidin. Snord oligoribonucleotide was treated as above, and the antibody-RNA complex was incubated with proteinase K for 30 min at 50°C to release the covalently bound RNA. The RNA was recovered by ethanol precipitation. cDNA was synthesized by Superscript II reverse transcriptase with a biotinylated primer (Biotin-T7) and a template-switching chimeric DNA:RNA oligonucleotide (SMAT oligo) to ensure the addition of an anchor sequence to the end of newly synthesized cDNA [24]. After 30 min of incubation at 37°C, the cDNA was recovered by streptavidin-coated magnetic beads (M-280 streptavidin Dynabeads, Thermo Fisher Scientific). PCR was performed using the Primestar DNA polymerase with T7-Nhe and SMAT S primer in a Bio-Rad MyCycler (40 cycles of 94°C for 30 seconds, 55°C for 30 seconds and 72°C for 10 seconds). The primer sequences are shown in S2 Table. The amplified DNA was digested with *Nhe*I and subcloned into pBluescript. The nucleotide sequences were determined with an Applied Biosystems 373 DNA sequencer using a BigDye Terminator v.3.1 Cycle Sequence Kit (Applied Biosystems). The numbers of reverse transcription terminations (truncations) and single-nucleotide mismatches (transitions) were counted.

## Results

### Molecular cloning of mAbs against m6A-modified RNA

**Fig 1:**
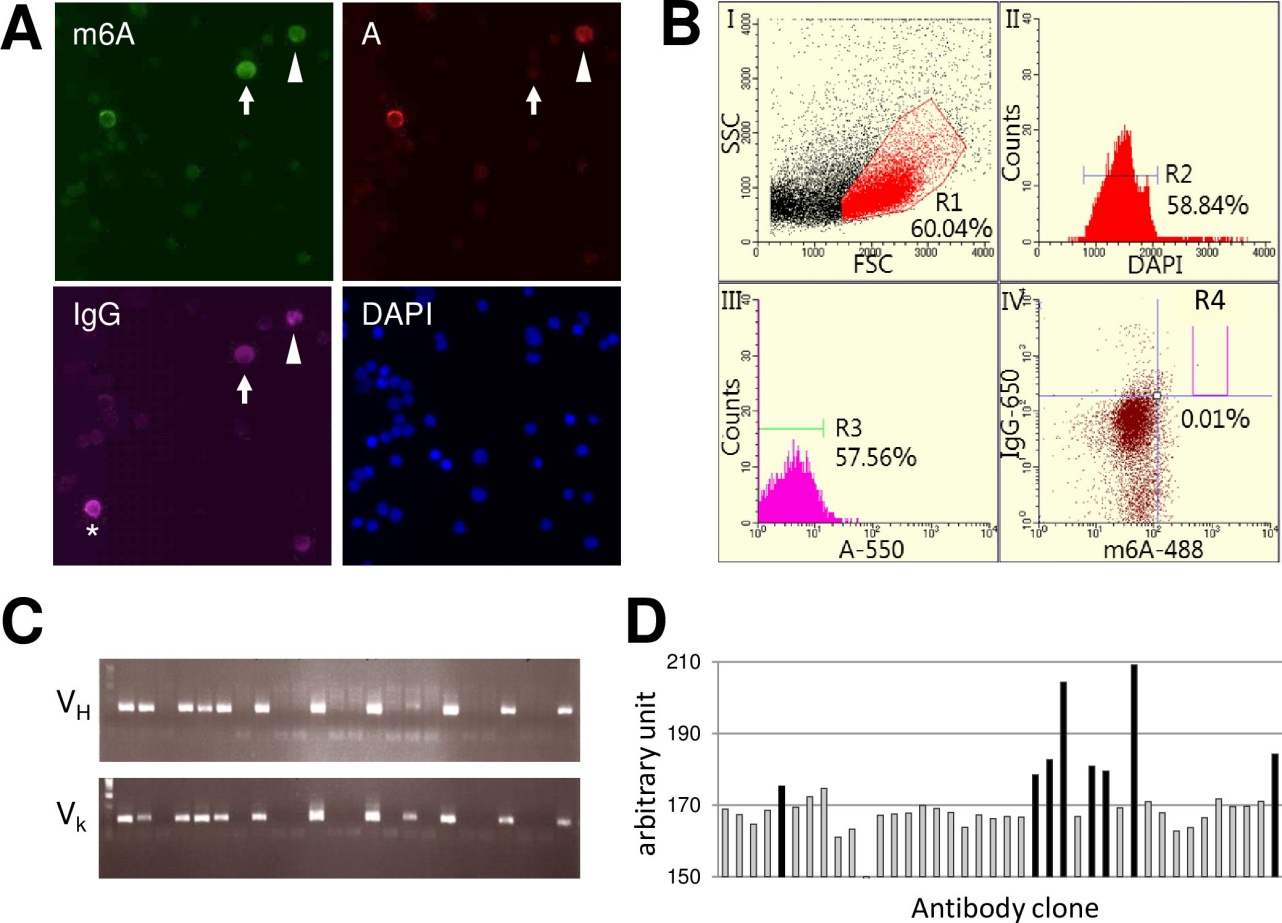
Generation of mAbs against m6A from immunized guinea pigs. (A) Fluorescence microscopy images of m6A-specific PCs. Iliac lymph node cells were fixed with paraformaldehyde-PBS and were intracellularly stained with m6A-oligo-488 (green), A-oligo-550 (red), IgG-650 (magenta) and DAPI (blue). Arrow: m6A-specific PC stained with m6A-oligo-488 and IgG-650 but not with A-oligo-550. Arrowhead: pan-A-specific PC stained with m6A-oligo-488, A-oligo-550 and IgG-650. Asterisk: nonspecific PC. (B) FACS gating strategy for the isolation of m6A-specific PCs by FIXAA. Cells treated as in (A) were subjected to FACS analysis. Plots (I) to (IV) represent the sequential gating strategy. (I) FSC vs SSC with gate R1 represents lymphocytes. (II) Single cells were selected via DAPI staining (R2). (III) Cells labeled with A-oligo-550 were excluded from the R2 gate (R3). (IV) The m6A-oligo-488^high^, A-oligo-550^negative^ and IgG-650^high^ fraction was defined as the m6A-specific PCs (R4). (C) Representative agarose gel electrophoresis of cognate pairs of V_H_ and V_L_ genes amplified from single cell-sorted R4-gated cells in (B). (D) In-cell ELISA screening of cells expressing anti-m6A mAbs. Cognate pairs of IgH and IgL genes were cotransfected into 293FT cells. The cells were fixed, permeabilized and stained with m6A-oligo-488, A-oligo-550 and IgG-650 and then subjected to imaging analysis. The intensity of m6A-oligo-488 relative to that of A-oligo-550 in IgG positive cells was determined. Antibody clones with black columns were selected.

m6A is not immunogenic by itself but can elicit an immune response via coupling to an immunogenic carrier protein. To elicit immune responses, we conjugated m6A-oligo to KLH and immunized guinea pigs with it. To analyze the presence of m6A-specific PCs in the animals, lymph node cells were fixed and stained intracellularly with biotinylated m6A-oligo conjugated to DyLight 488 streptavidin (m6A-oligo-488), biotinylated A-oligo conjugated to DyLight 550 streptavidin (A-oligo-550) and DyLight 650 anti-guinea pig IgG (IgG-650). Fluorescence microscopy analysis revealed that a very limited number of PCs (less than 0.002% of the total lymph node cells) were intensely labeled with m6A-oligo-488 and IgG-650 but not with A-oligo-550, indicating the presence of m6A-specific PCs in immunized animals (Fig 1A).

To isolate m6A-specific PCs, we applied a FACS-based antigen-specific PC separation method, FIXAA, which targets abundant intracellular immunoglobulin to tag fluorescently labeled antigens. The positive selection of PCs with m6A-oligo-488 and negative selection with A-oligo-550 revealed a fraction of m6A-specific PCs (R4-gate) that were defined as m6A-oligo-488^high^, A-oligo-550^negative^ and IgG-650^high^ (Fig 1B). Single cell-based 5′-RACE PCR of the R4-gated cells resulted in the amplification of cognate pairs of V_H_ and V_L_ (Fig 1C). After the preparation of IgH and IgL chain constructs by TSj-PCR, each pair of immunoglobulin genes was transfected into 293FT cells grown in a 96-well plate to express recombinant antibody. Two days after the transfection, in-cell ELISA screening was performed by intracellularly staining the expressed antibodies with m6A-oligo-488, A-oligo-550 and IgG-650 (S1 Fig). Among the forty clones screened, eight clones specifically reacted with m6A-oligo-488 but not with A-oligo-550 (Fig 1D).

### #B1-3 recognizes a single m6A in oligoribonucleotide

**Fig 2:**
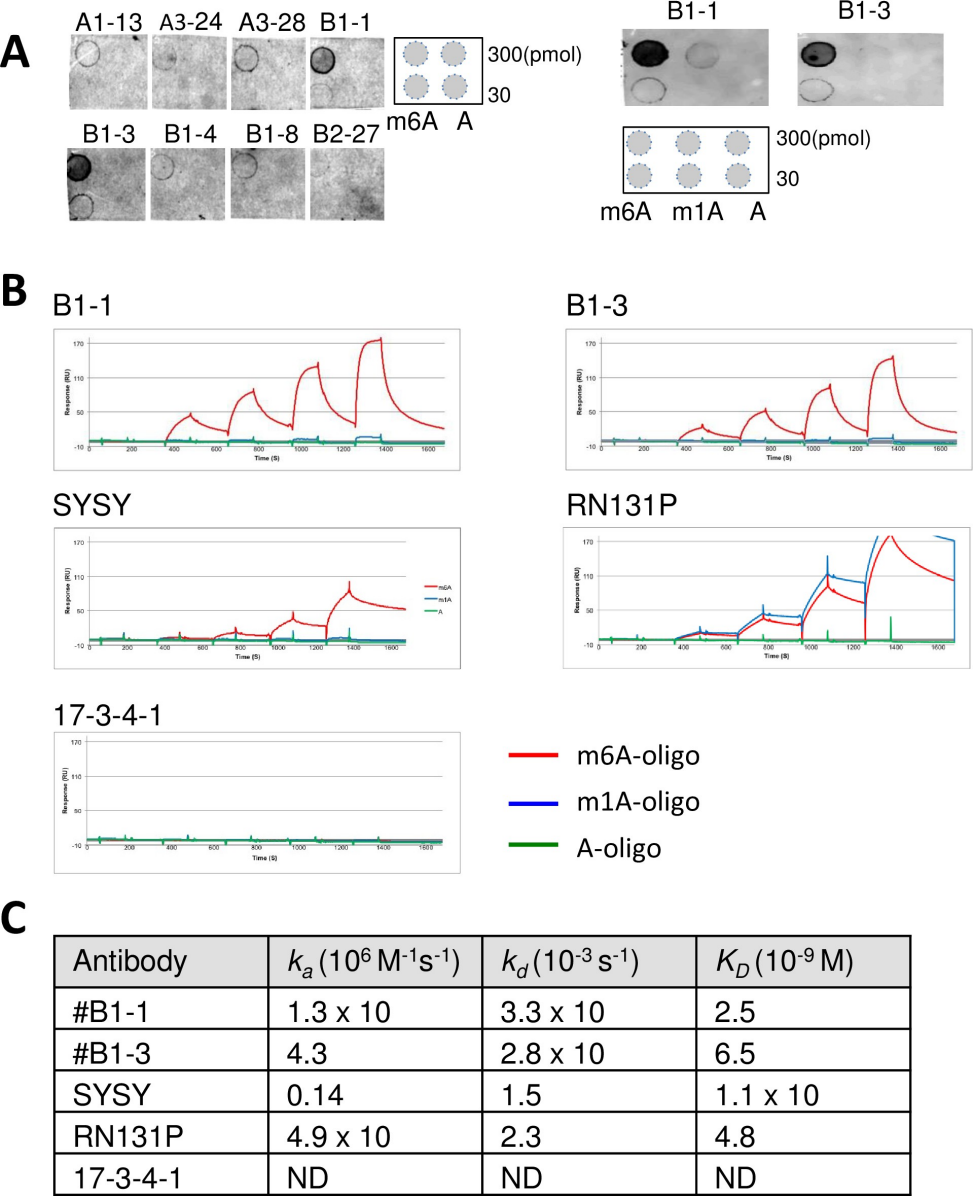
Antibody screening by dot blotting and SPR. (A) Dot blot screening of mAb clones. The indicated amount of oligoribonucleotides were spotted onto membranes and probed with mAb clones. (B) Antibody affinity determination by SPR. Biotinylated oligonucleotides were immobilized on streptavidin sensor chips, and antibodies were allowed to bind at different dilutions (0 ~ 27 nM). A representative SPR sensorgrams are shown. (C) The association and dissociation rate constants (ka and kd) and equilibrium constants (K_D_) obtained from (B) are shown.

Next, we expressed the eight mAb clones in Expi293 cells, and the purified mAbs were used for dot blot analysis, in which increasing amounts of RNA oligonucleotides were immobilized on a nitrocellulose membrane. The top two clones (#B1-1 and #B1-3) strongly reacted with m6A-oligo but not with A- or m1A-oligo (Fig 2A).

To characterize the antibody-antigen interactions, SPR analysis was performed with commercially available rabbit polyclonal antibodies (SYSY and RN131P) and a mouse mAb (17-3-4-1). #B1-1 and #B1-3 specifically reacted with m6A-oligo (equilibrium dissociation constant (K_D_) = 2.5 nM and 6.5 nM, respectively) but not with m1A-oligo or A-oligo. SYSY also showed specific binding, but RN131P cross-reacted with m1A-oligo. 17-3-4-1 failed to bind to m6A-oligo (Fig 2B and 2C). We selected #B1-3 and further validated its specificity by ELISA. #B1-3 showed dose–dependent binding to immobilized m6A-oligo but exhibited negligible binding to m1A- or A-oligo (S2 Fig). Competitive ELISA performed with m6ATP or m6-adenine at 1 μM completely abolished #B1-3 binding to immobilized m6A-oligo, but ~1,000 times as much ATP or m1ATP was needed for equivalent competition. Increasing concentrations of free m6A-oligo progressively attenuated #B1-3 binding to immobilized m6A-oligo, but increasing concentrations of free m1A- or A-oligo did not affect binding. To analyze whether this antibody can be used for immunoprecipitation, #B1-3 was mixed with biotinylated m6A-oligo, m1A-oligo or A-oligo, and oligoribonucleotide-antibody complexes were recovered with protein A beads. As shown in S3 Fig, #B1-3 specifically immunoprecipitated m6A-oligo with 35% efficiency.

### #B1-3 detects endogenous m6A in RNA

**Fig 3:**
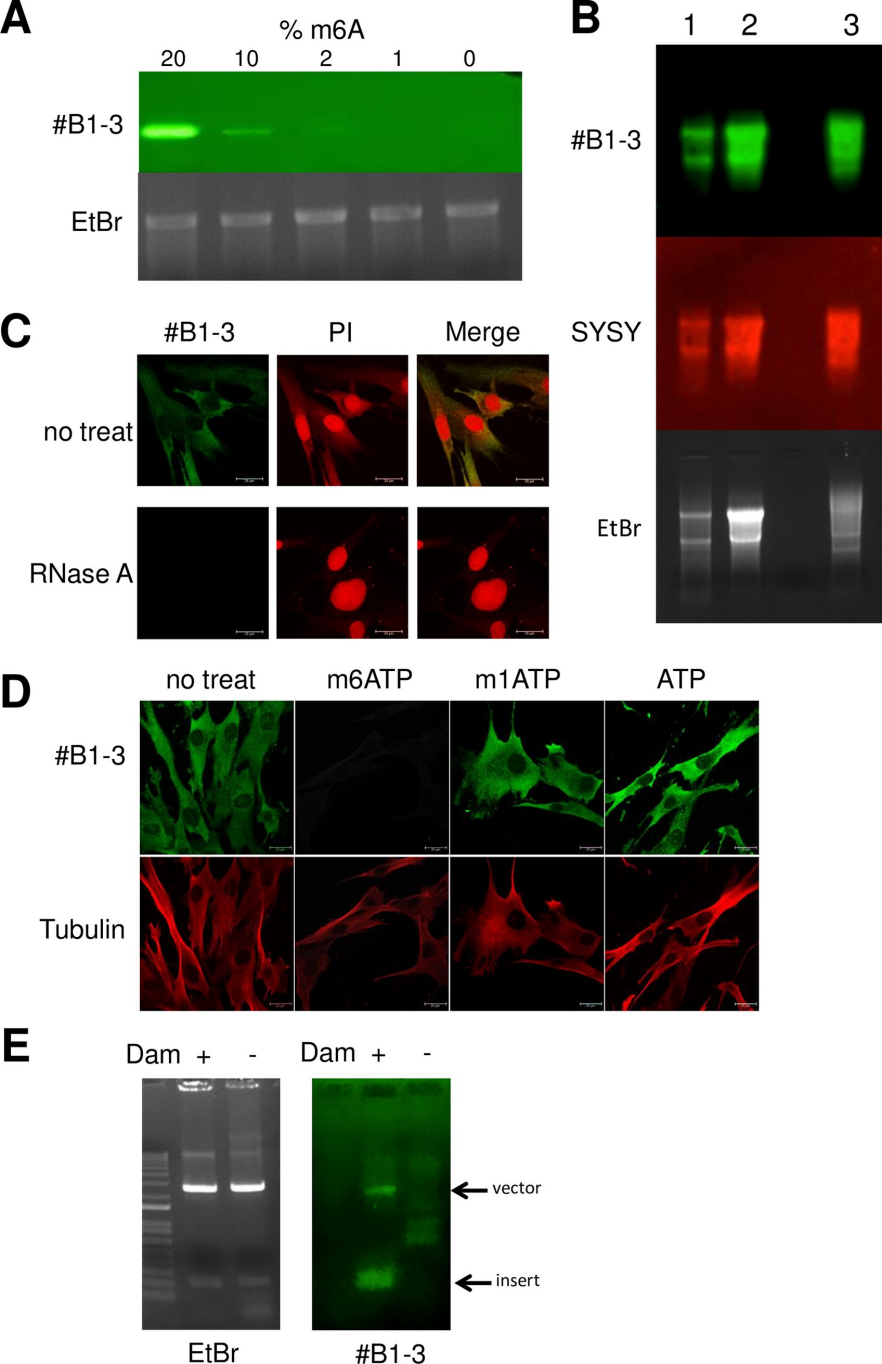
#B1-3 detects endogenously expressed m6A-modified RNA. (A) Immuno-Northern blot detection of m6A in *in vitro*-transcribed RNA. Aliquots (0.1 μg) of *in vitro*-transcribed RNA with or without m6A modification were separated on denaturing agarose gels. The RNA blotted on the nitrocellulose membrane was subjected to immunoblotting with #B1-3. The m6ATP/ATP ratio (%) in the RNA is indicated. (B) Immuno-Northern blot detection of m6A in RNA extracted from cells. Poly(A)-enriched RNA from human spleen (lane 1), Jurkat cells (lane 2) and placenta (lane 3) was treated as in (B). The signals detected by #B1-3 and SYSY are shown. Ethidium bromide (EtBr) staining of RNA is shown as a loading control. (C) Immunohistochemical detection of m6A in fibroblasts. Human fibroblasts pretreated with or without RNase A were stained with #B1-3. The specificity of RNA digestion was monitored by propidium Iodide (PI) staining. (D) Human fibroblasts were stained with #B1-3 (green) and tubulin (red) in the presence of 1 μM of m6ATP, m1ATP or ATP. (E) Immuno-Southern blot detection of DNA extracted from *E*.*coli*. Plasmids isolated from *dam*^+^ or *dam*^-^ *E*. *coli* were digested with *Eco*RI and separated with 1% agarose gel electrophoresis. The DNA blotted on nitrocellulose membranes was subjected to immunoblotting with #B1-3. EtBr staining of DNA is shown as a loading control.

We then used this antibody to carry out Immuno-northern blotting assays with *in vitro* transcribed RNA, in which 0 ~ 20% of adenosines were replaced by m6A. The enhanced signal according to the m6A content was detected by #B1-3. This antibody did not bind to unmodified RNA (Fig 3A).

To examine whether #B1-3 can detect endogenously expressed m6A in RNA, poly(A)-enriched RNA from human cells was subjected to Immuno-Northern blotting. #B1-3 detected m6A in all RNA samples tested, including ribosomal RNA and mRNA. The staining pattern was similar to that of SYSY antibody (Fig 3B). We also performed immunohistochemical staining with human fibroblasts; #B1-3 identified punctate structures throughout the cytosol. But #B1-3 signal was not detected on the cells treated with RNase A, indicating the specificity of #B1-3 for m6A in RNA (Fig 3C). The specificity of this mAb for m6A in RNA was also confirmed by competition assays, in which #B1-3 was incubated with m6ATP, m1ATP or ATP. Although incubation with either m1ATP or ATP did not affect the detection of m6A localization in cells, incubation with m6ATP caused an almost complete loss of reactivity (Fig 3D). *E*. *coli* Dam methylase introduces a methyl group to N6 position of deoxyadenosine at 5'-GATC-3' motif. To examine whether #B1-3 also binds to N6-methyl-2′-deoxyadenosine (m6dA) in which the −OH group on carbon 2 of ribose is replaced by hydrogen, we amplified plasmids containing DNA fragments carrying Dam methylation sites in either the *dam*+ or *dam*- *E*. *coli* strain and subjected to Immuno-Southern blotting [3]. #B1-3 bound to the DNA fragment obtained from the *dam*^*+*^ *E*. *coli* strain but failed to bind to the fragment from the *dam*^-^ *E*. *coli* strain (Fig 3E). These results indicate that #B1-3 specificity is dependent on the presence of methylation in an adenosine base at the nitrogen-6 position.

### #B1-3 induced truncations at the m6A position during reverse transcription

**Fig 4:**
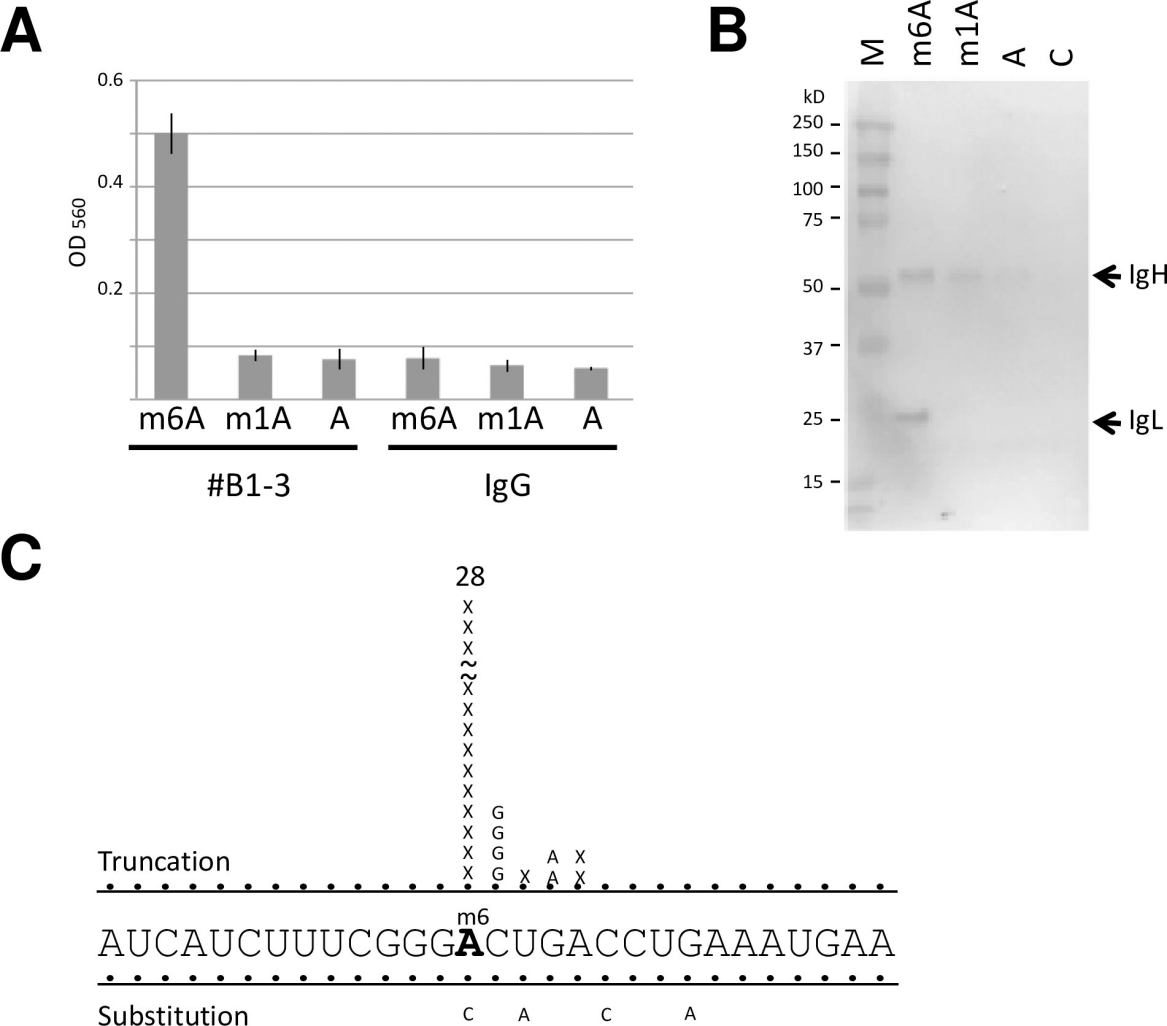
#B1-3 induces truncations in cDNA at m6A positions. (A) #B1-3 was UV-crosslinked to Biotinylated oligoribonucleotides and immunoprecipitated. The covalently bound oligoribonucleotides to the antibody were quantified with alkaline phosphatase-conjugated streptavidin. Relative binding are represented as the average ± SD of three replicates. (B) The antibody-oligoribonucleotides complexes in (A) were separated by SDS-PAGE, transferred to nitrocellulose membranes and probed by Western blotting with alkaline phosphatase-conjugated streptavidin. (C) cDNA was synthesized from Snord-oligo recovered from UV-crosslinked antibody-oligoribonucleotides complex. 5’-RACE PCR amplified DNA was subcloned into plasmid and sequenced. The frequencies of positions with crosslinking-induced truncations (denoted as X) and nucleotide substitutions are shown.

We next tried to determine whether #B1-3 could be used for miCLIP. To test this possibility, #B1-3 was mixed with biotinylated m6A-oligo, m1A-oligo or A-oligo, UV irradiated and then immunoprecipitated with Protein A magnetic beads. As shown in Fig 4A, this antibody specifically immunoprecipitated m6A-oligo but not m1A- or A-oligo. Control IgG did not immunoprecipitate m6A-oligo.

To analyze the formation of covalent bonds between oligoribonucleotides and the antibody by UV irradiation, the immunoprecipitates were separated with SDS-PAGE, transferred to membranes, and then probed with streptavidin. It was found that the light chain of #B1-3 was predominantly crosslinked with m6A-oligo but not with m1A- or A-oligo (Fig 4B). We then used this antibody to carry out miCLIP with a 34-nt Snord2 oligoribonucleotide (Snord-oligo) containing a single m6A. #B1-3 was UV-crosslinked to biotinylated Snord-oligo, immunoprecipitated and purified after Portease K treatment. Recovered Snord-oligo was reverse-transcribed, and cDNA amplified by 5’-RACE PCR was subcloned into plasmid. When a total of 41 individual clones were sequenced to identify the nucleotide substitutions and/or truncations created during reverse transcription, we found that #B1-3 efficiently induced truncations at the m6A position with 68% efficiency, while nucleotide substitutions occurred with reduced positional accuracy (Fig 4C).

### Development of a mAb against pseudouridine

Pseudouridine (ΨU) is present in a wide variety of cellular RNAs and is highly conserved across species [25,26]. To validate the application of our technology to the development of mAbs against modified nucleotides, we attempted to generate mAbs specific for ΨU. As shown in S4 Fig, the screening of PCs from guinea pigs immunized with oligoribonucleotides containing a single ΨU resulted in the generation of a mAb that specifically bound to ΨU in RNA.

## Discussion

Modified nucleotides are non-immunogenic small molecules that differ only slightly in terms of their chemical structure from unmodified molecules. Thus, it is extremely difficult to produce mAbs with high affinity and specificity against modified nucleotides. In this paper, we used our recently developed FACS-based antigen-specific PC isolation method, FIXAA, which can efficiently remove cross-reactive or unspecific PCs before primary antibody screening. This resulted in the efficient generation of guinea pig mAbs that can recognize single m6A in RNA without cross-reacting with m1A and unmodified adenosine.

In addition to internal m6A, m6Am is present at the transcription start site in capped mRNAs [27]. We used m6A-oligo for coupling to KLH, which allowed the ribose molecule in m6A to remain intact. This oligoribonucleotide–protein conjugate potentially allows the generation of mAbs that discriminate deoxyribose, ribose and ribose with 2’-O-methylation. Our results showed that #B1-3 bound to m6A in RNA and m6dA in DNA and that the m6A binding was inhibited by m6ATP and m6-adenine but not by m1ATP or ATP, suggesting that sugar-phosphate backbone does not act as the antigenic determinant of this antibody. However, it is not clear whether #B1-3 recognizes m6Am. Further analysis will be needed to clarify this point.

Although recent technological advances have enabled the transcriptome-wide identification of m6A, the validity of the identification depends on the specificity and affinity of the antibody used. Currently, several polyclonal and monoclonal antibodies against m6A have been used to target m6A to specifically detect and/or enrich m6A-modified RNA. To serve as reliable research tools, these clones need to be thoroughly validated in terms of their affinity and specificity in the context of their intended use. Comparison of the binding specificity and affinity of #B1-3 to those of commercially available anti-m6A antibodies by SPR revealed a remarkable difference in the detection of m6A-oligo. #B1-3 and SYSY can detect single m6A nucleotides, but 17-3-4-1 failed to bind it. This suggests that 17-3-4-1 needs more than a single m6A nucleotide in RNA for efficient binding. Although #B1-3 and SYSY displayed similar K_D_ values, their binding kinetics are different; #B1-3 displayed a 100-fold higher association rate (ka) but a 10-fold higher dissociation rate (kd) than SYSY. This indicates that #B1-3 can form immune complexes faster, but the antibody-antigen bond breaks more easily. To perform more sensitive detection of m6A, *in vitro* affinity maturation, using phage display technique, will be required. It has been shown that C→T transitions are a common result of the use of Abcam antibody in miCLIP [27]. However, #B1-3 primarily induced truncations at the m6A position during reverse transcription, while C→T substitutions at the +1 position were not observed. The differences in the mutational patterns in cDNAs are probably due to differences in the sets of amino acid residues that make contact with m6A. These results suggest that the observed truncation might be used as a mutational signature of this antibody to map the m6A nucleotides in RNA. However, since various factors induce cDNA truncations which lead false positive signals, care will be taken when using #B1-3 to perform miCLIP.

Collectively, our data demonstrate the high sensitivity and specificity of the anti-m6A mAb for detecting m6A-modified RNA and validate the feasibility of our technology for generating mAbs against modified nucleosides.

## Supporting information

S1 Table Oligoribonucleotide sequence used in this experiment. (DOCX)

S2 Table Primer sequences used for miCLIP. (DOCX)

S1 File Nucleotide sequence of DNA containing 11 Dam methylation sites. (DOCX)

S1 Fig Antibody screening by in-cell ELISA. (A) Flow chart of in-cell ELISA. Antibody-expressing 293FT cells were fixed and membrane permeabilized. The cells were stained with m6A-oligo-488, A-oligo-550 and IgG-650 and analyzed by an imaging cytometer.
(B) Representative images of cells expressing m6A-specific antibody are shown.
(PDF)

S2 Fig Analysis of #B1-3 binding by ELISA. (A) Dose-dependent binding of #B1-3 to m6A-oligo. #B1-3 was serially diluted and tested by ELISA for antibody binding to m6A-, m1A- or A-oligo (n = 2).
(B) Nucleotide competition experiments performed by ELISA. The indicated blocking nucleotides (0~1 mM) were preincubated with #B1-3. The antibody mixtures were applied to wells with immobilized m6A-oligo (n = 3).
(C) RNA oligonucleotide competition experiments performed by ELISA. The indicated blocking oligoribonucleotides (0~1 μM) were preincubated with #B1-3. The antibody mixtures were applied to wells with immobilized m6A-oligo (n = 2). The relative binding indicates the ratio of the ELISA signal in the presence of the competitor to that in the absence of the competitor. Each experiment was repeated twice and data from a single representative experiment are presented.
(D) m6-adenine (0~1 mM) was preincubated with #B1-3, and the antibody mixtures were applied to wells with immobilized m6A-oligo (n = 2).
(PDF)

S3 Fig #B1-3 immunoprecipitates oligoribonucleotide containing m6A. (A) Each 4 μg of #B1-3 or control IgG was mixed with 2μM of biotinylated m6A-, m1A- or A-oligo in 20 μl of PBST and incubated for 15 min at room temperature. After addition of protein A Dynabeads, antibody-captured oligoribonucleotides were recovered in pellet. The pellets were washed with PBST for three times, and the antibody-captured oligoribonucleotides were quantified with alkaline phosphatase-conjugated streptavidin. Relative binding are represented as the average ± SD of three replicates.
(B) The efficiency of immunoprecipitation was calculated by dot-blot analysis. The pellets in (A) were suspended in 20 μl of PBST containing 0.1mM of m6ATP and incubated for 15 min at 37°C to elute m6A-oligo. The input and the eluate were blotted on nitrocellulose membranes, and the amount of m6A-oligo was quantified with alkaline phosphatase-conjugated streptavidin.
(PDF)

S4 Fig Generation of pseudouridine-specific mAbs. (A) Fluorescence microscopy images of pseudouridine (ΨU)-specific PCs. Iliac lymph node cells immunized with KLH-conjugated ΨU-oligoribonucleotide (ΨU-oligo, rNrNrNrNrN/ΨU/rNrNrNrN) were fixed with paraformaldehyde-PBS and intracellularly stained with ΨU-oligo-488 (green), U-oligo-550 (red), IgG-650 (magenta) and DAPI (blue). Arrow: ΨU-specific PC stained with ΨU-oligo-488 and IgG-650 but not U-oligo-550.
(B) FACS gating strategy for the isolation of ΨU-specific PCs by FIXAA. Cells treated as in (A) were subjected to FACS analysis. Plots (I) to (IV) represent the sequential gating strategy. (I) FSC vs SSC with gate R1 represents lymphocytes. (II) Single cells were selected via DAPI staining (R2). (III) Cells labeled with U-oligo-550 were excluded from the R2 gate (R3). (IV) The ΨU-oligo-488^high^, U-oligo-550^negative^ and IgG-650^high^ fraction was defined as the m6A-specific PCs (R4).
(C) Immuno-Northern blot detection of ΨU-modified RNA. Aliquots (0.1 μg) of *in vitro*-transcribed RNA with or without ΨU-modifications were analyzed on denaturing agarose gels followed by EtBr staining and UV illumination (upper). The RNAs blotted on nitrocellulose membranes were subjected to immunoblotting with antibody clone #B3-19 (bottom). #B3-19 mAb bound to ΨU-modified RNA but not to unmodified RNA. The ΨU content (%) in the RNA is indicated.
(D) Dose-dependent binding of #B3-19 to ΨU-oligo. #B3-19 was serially diluted and tested by ELISA for antibody binding to ΨU-oligo or unmodified-oligo.
(PDF)

S1 Raw Images (ZIP)
