# Supplementary material for: Development and validation of monoclonal antibodies against N6-methyladenosine for the detection of RNA modifications
Source: PLoS One. 2019 Oct 2;14(10):e0223197. doi: 10.1371/journal.pone.0223197 (PMC6774519; doi:10.1371/journal.pone.0223197)
Supplement: S1 Table — (DOCX) [file pone.0223197.s001.docx]

**S1 Table. Oligoribonucleotides used in this experiment.**

| m6A-oligo (Biotin)-rNrNrNrGrG/m6rA/rCrNrNrN  m1A-oligo (Biotin)-rNrNrNrGrG/m1A/rCrNrNrN  A-oligo (Biotin)-rNrNrNrGrG/rA/rCrNrNrN  Snord oligo rArUrCrArUrCrUrUrUrCrGrGrG/m6rA/rCrUrGrArCrCrUrGrArArArUrGrArArCrCrCrUrA |
| --- |
| N: any nucleotide  Underline: T7 primer annealing site |
