# Supplementary material for: Development and validation of monoclonal antibodies against N6-methyladenosine for the detection of RNA modifications
Source: PLoS One. 2019 Oct 2;14(10):e0223197. doi: 10.1371/journal.pone.0223197 (PMC6774519; doi:10.1371/journal.pone.0223197)
Supplement: S2 Table — (DOCX) [file pone.0223197.s002.docx]

**S2 Table. Primer sequences used for miCLIP**

| Biotin-T7 Biotin-TAATACGACTCACTATAGGG  SMAT oligo ACC(SpacerC3)CGCTAGCGCTACCGGACTCAGATGrGrGrG  SMAT S1 GCTAGCGCTAGCGCTACCGGACTCAGATGGGG  T7-Nhe AS GAGAGAGAGCTAGCTAATACGACTCACTATAGGG |
| --- |
|  |
