# Supplementary material for: Development and validation of monoclonal antibodies against N6-methyladenosine for the detection of RNA modifications
Source: PLoS One. 2019 Oct 2;14(10):e0223197. doi: 10.1371/journal.pone.0223197 (PMC6774519; doi:10.1371/journal.pone.0223197)
Supplement: S1 File — (DOCX) [file pone.0223197.s003.docx]

**S1 File. Nucleotide sequence of DNA containing 11 Dam methylation sites.**

GAATTCAGATAATACGACTCACTATAGGGCTCTGTGTTCTGTTCGATCGTCCTGCGATCTTCTTTTCGCTGATCGCTGCGCGGCTTGATCGTCCTGTTCCTGGATCTGTCCTGTTCGATCTAGATCTTGTCTGTCCTGTTGTTGATCTCTTTGTTCCTGATCGTGTGTCTCCCTGATCGTGTTGCTGTTGATCTCCTTCCTGTGCGTTGTTGCTTCGTTCGCCTGTCGTGTTCCTCGAGGAATTC

Underlining indicates Dam methylation sites.
