# Supplementary figures and images for: Development and validation of monoclonal antibodies against N6-methyladenosine for the detection of RNA modifications
Source: PLoS One. 2019 Oct 2;14(10):e0223197. doi: 10.1371/journal.pone.0223197 (PMC6774519; doi:10.1371/journal.pone.0223197)

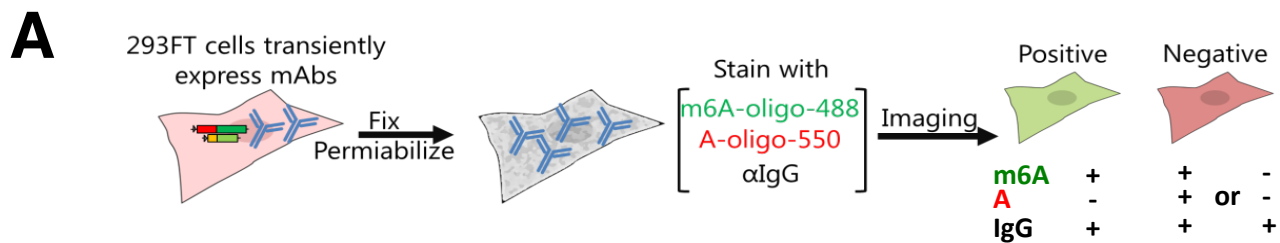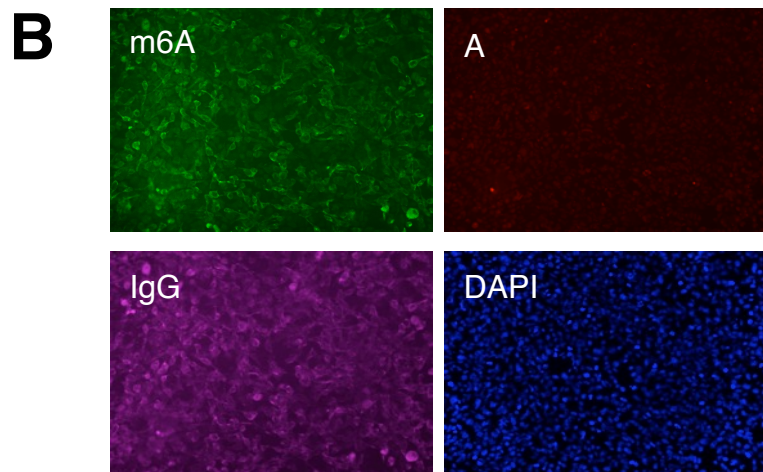

Supplement: S1 Fig — (A) Flow chart of in-cell ELISA. Antibody-expressing 293FT cells were fixed and membrane permeabilized. The cells were stained with m6A-oligo-488, A-oligo-550 and IgG-650 and analyzed by an imaging cytometer. (B) Representative images of cells expressing m6A-specific antibody are shown. (PDF) [file pone.0223197.s004.pdf]

**A**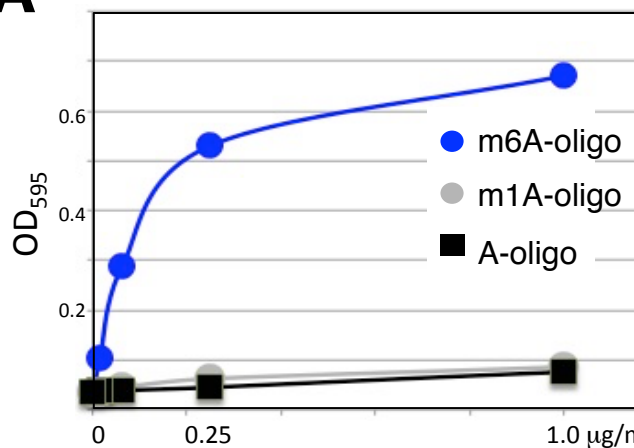**B**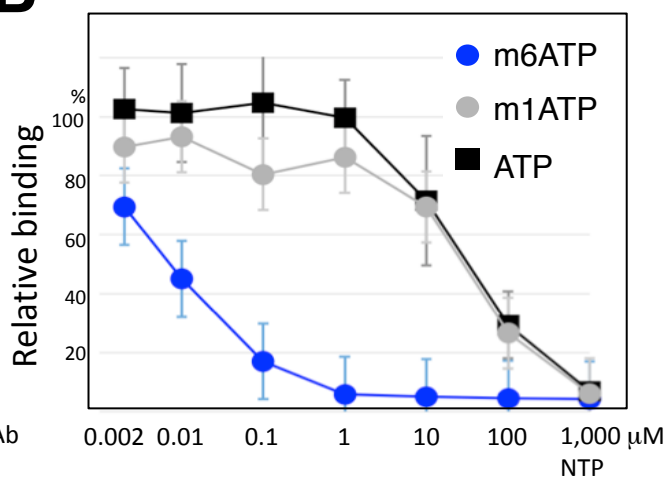**C**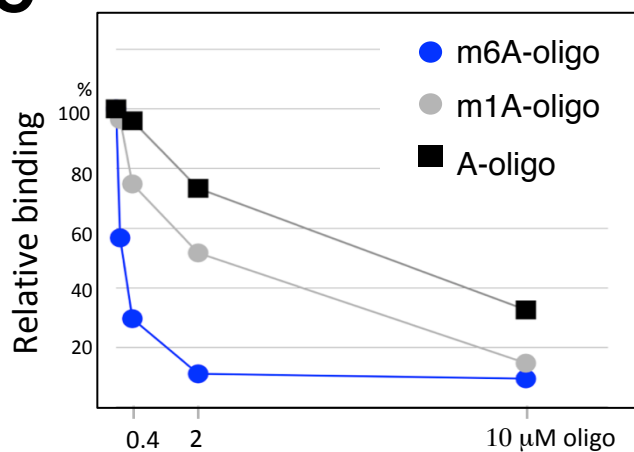**D**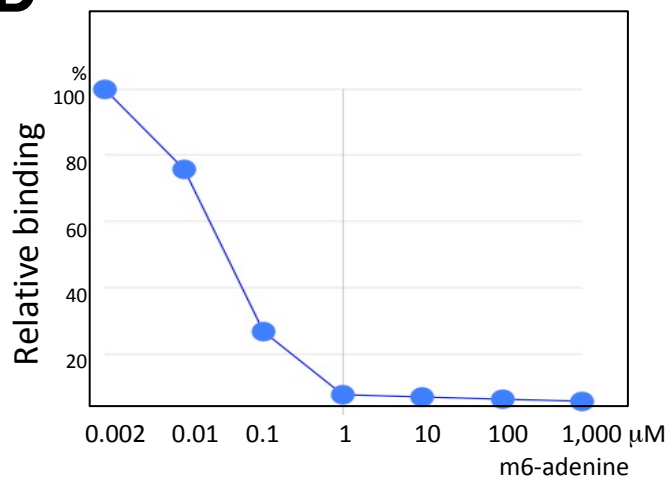

Supplement: S2 Fig — (A) Dose-dependent binding of #B1-3 to m6A-oligo. #B1-3 was serially diluted and tested by ELISA for antibody binding to m6A-, m1A- or A-oligo (n = 2). (B) Nucleotide competition experiments performed by ELISA. The indicated blocking nucleotides (0~1 mM) were preincubated with #B1-3. The antibody mixtures were applied to wells with immobilized m6A-oligo (n = 3). (C) RNA oligonucleotide competition experiments performed by ELISA. The indicated blocking oligoribonucleotides (0~1 μM) were preincubated with #B1-3. The antibody mixtures were applied to wells with immobilized m6A-oligo (n = 2). The relative binding indicates the ratio of the ELISA signal in the presence of the competitor to that in the absence of the competitor. Each experiment was repeated twice and data from a single representative experiment are presented. (D) m6-adenine (0~1 mM) was preincubated with #B1-3, and the antibody mixtures were applied to wells with immobilized m6A-oligo (n = 2). (PDF) [file pone.0223197.s005.pdf]

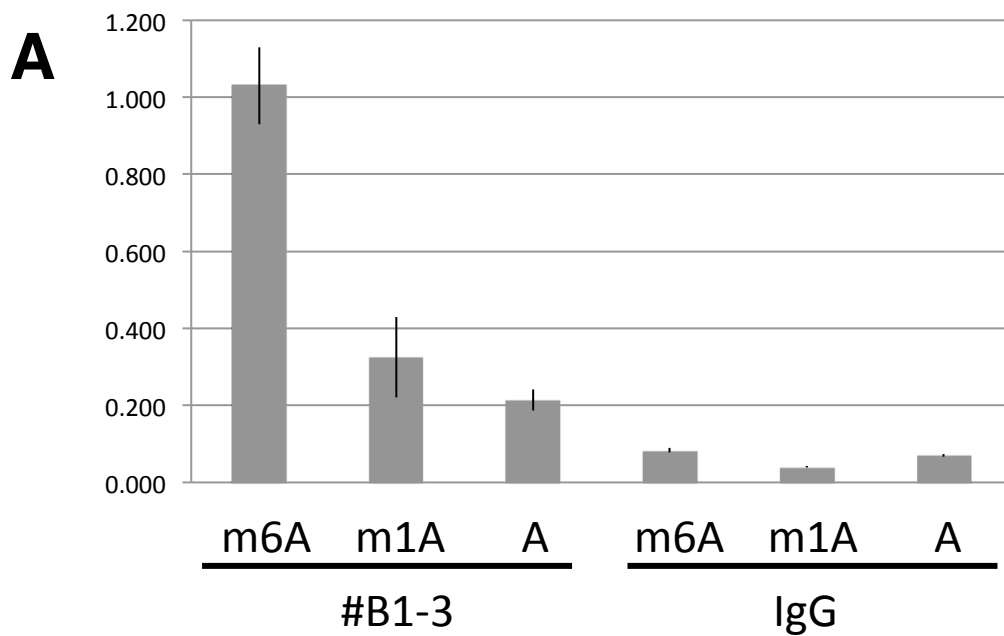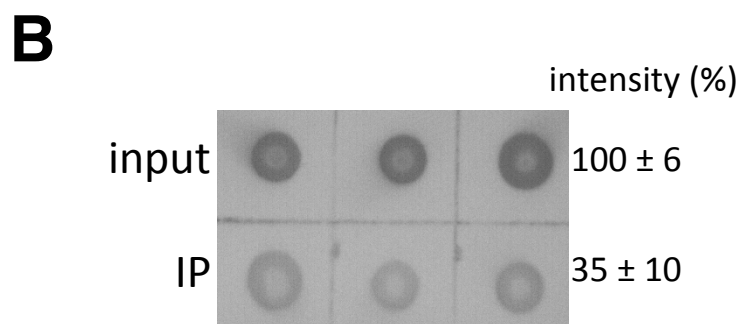

Supplement: S3 Fig — (A) Each 4 μg of #B1-3 or control IgG was mixed with 2μM of biotinylated m6A-, m1A- or A-oligo in 20 μl of PBST and incubated for 15 min at room temperature. After addition of protein A Dynabeads, antibody-captured oligoribonucleotides were recovered in pellet. The pellets were washed with PBST for three times, and the antibody-captured oligoribonucleotides were quantified with alkaline phosphatase-conjugated streptavidin. Relative binding are represented as the average ± SD of three replicates. (B) The efficiency of immunoprecipitation was calculated by dot-blot analysis. The pellets in (A) were suspended in 20 μl of PBST containing 0.1mM of m6ATP and incubated for 15 min at 37°C to elute m6A-oligo. The input and the eluate were blotted on nitrocellulose membranes, and the amount of m6A-oligo was quantified with alkaline phosphatase-conjugated streptavidin. (PDF) [file pone.0223197.s006.pdf]

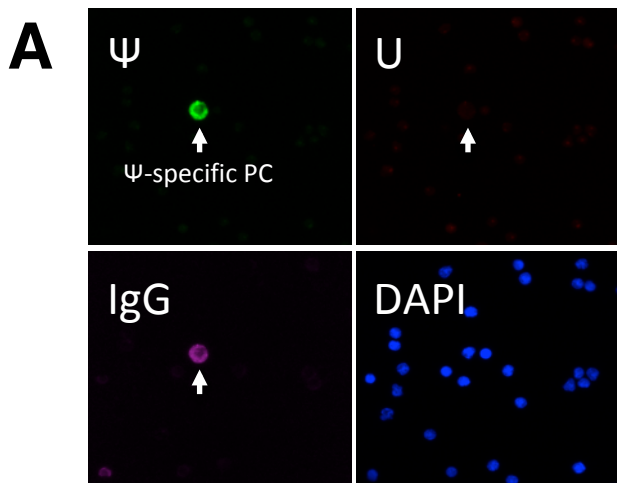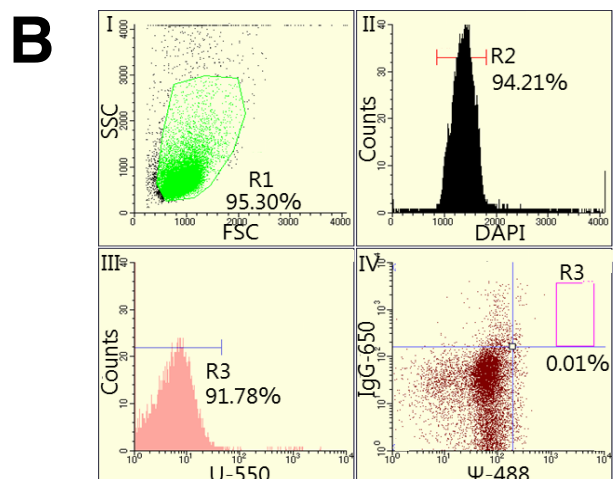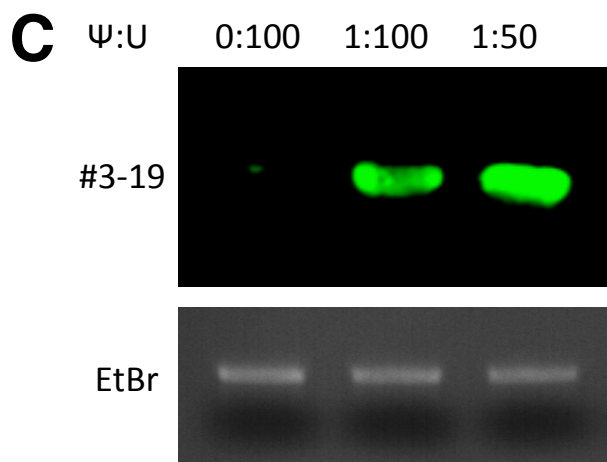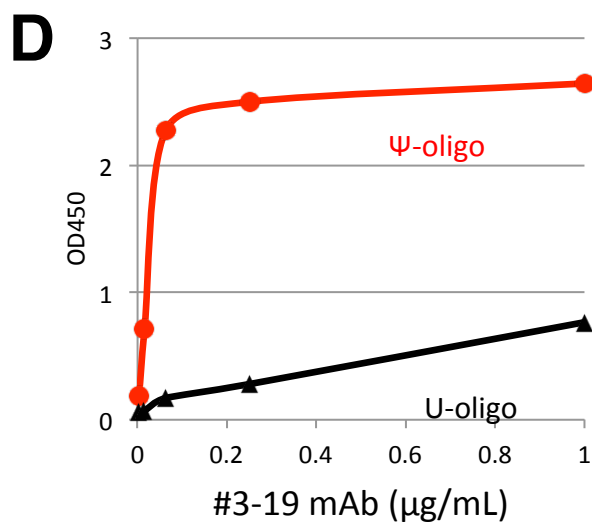

Supplement: S4 Fig — (A) Fluorescence microscopy images of pseudouridine (ΨU)-specific PCs. Iliac lymph node cells immunized with KLH-conjugated ΨU-oligoribonucleotide (ΨU-oligo, rNrNrNrNrN/ΨU/rNrNrNrN) were fixed with paraformaldehyde-PBS and intracellularly stained with ΨU-oligo-488 (green), U-oligo-550 (red), IgG-650 (magenta) and DAPI (blue). Arrow: ΨU-specific PC stained with ΨU-oligo-488 and IgG-650 but not U-oligo-550. (B) FACS gating strategy for the isolation of ΨU-specific PCs by FIXAA. Cells treated as in (A) were subjected to FACS analysis. Plots (I) to (IV) represent the sequential gating strategy. (I) FSC vs SSC with gate R1 represents lymphocytes. (II) Single cells were selected via DAPI staining (R2). (III) Cells labeled with U-oligo-550 were excluded from the R2 gate (R3). (IV) The ΨU-oligo-488high, U-oligo-550negative and IgG-650high fraction was defined as the m6A-specific PCs (R4). (C) Immuno-Northern blot detection of ΨU-modified RNA. Aliquots (0.1 μg) of in vitro-transcribed RNA with or without ΨU-modifications were analyzed on denaturing agarose gels followed by EtBr staining and UV illumination (upper). The RNAs blotted on nitrocellulose membranes were subjected to immunoblotting with antibody clone #B3-19 (bottom). #B3-19 mAb bound to ΨU-modified RNA but not to unmodified RNA. The ΨU content (%) in the RNA is indicated. (D) Dose-dependent binding of #B3-19 to ΨU-oligo. #B3-19 was serially diluted and tested by ELISA for antibody binding to ΨU-oligo or unmodified-oligo. (PDF) [file pone.0223197.s007.pdf]
