# Supplementary material for: Development and validation of monoclonal antibodies against N6-methyladenosine for the detection of RNA modifications
Source: PLoS One. 2019 Oct 2;14(10):e0223197. doi: 10.1371/journal.pone.0223197 (PMC6774519; doi:10.1371/journal.pone.0223197)
Supplement: S1 Raw Images — (ZIP) [file pone.0223197.s008.zip › Plod row data.pdf]

Raw Fig 1A

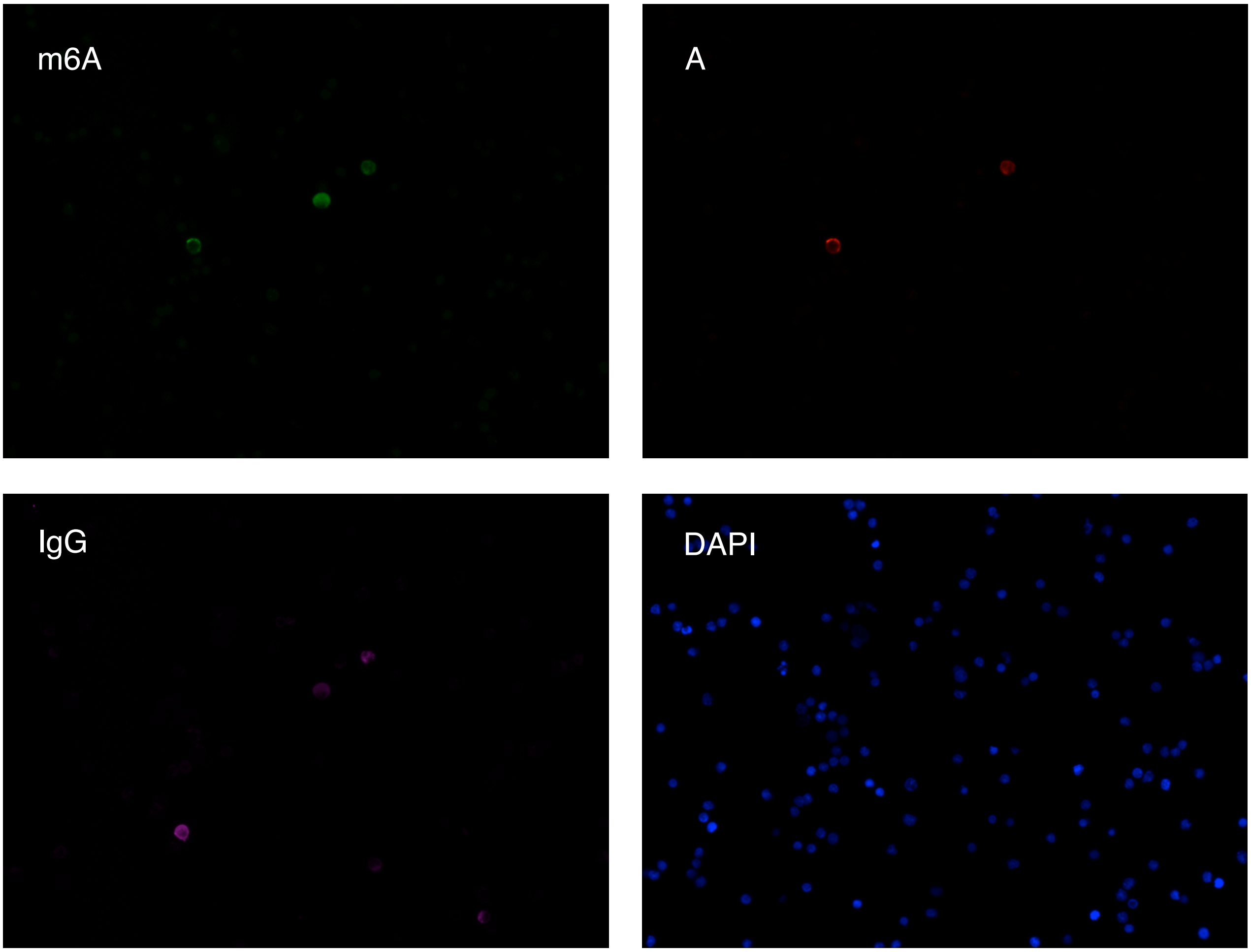

Raw Fig.1C

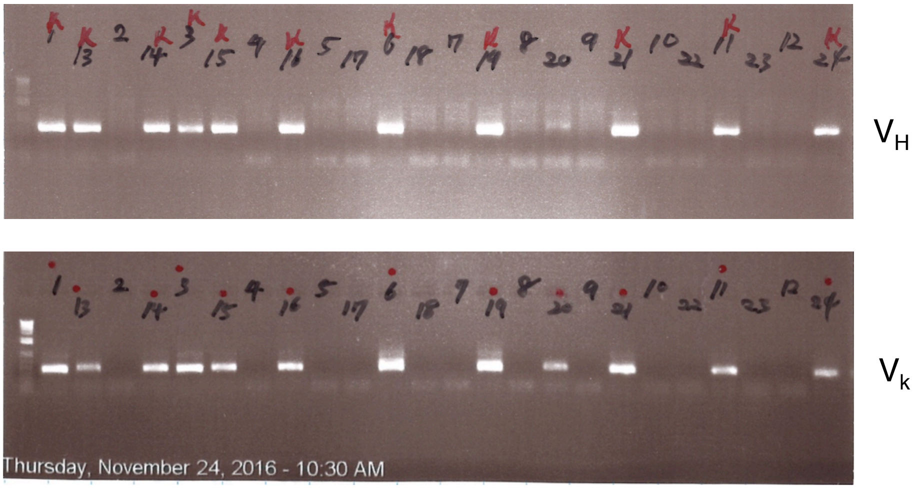

Raw Fig 2A

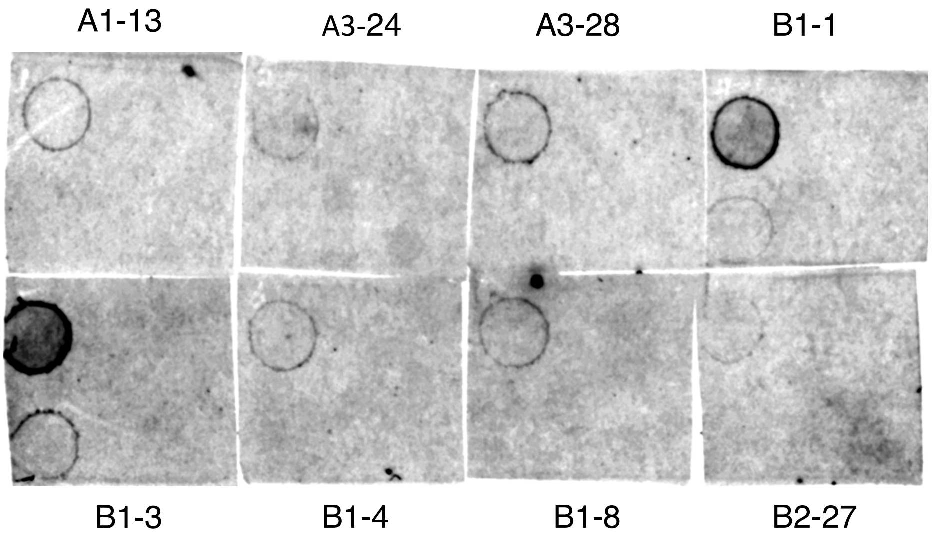

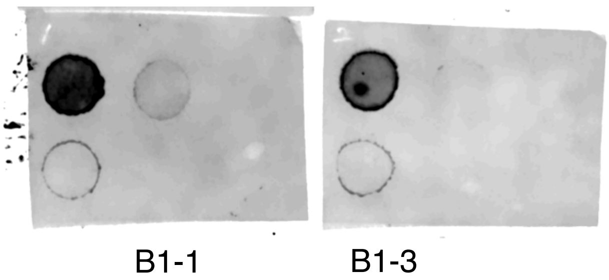

Raw Fig 3A

#B1-3

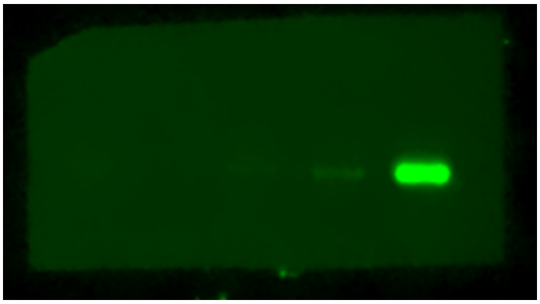

EtBr

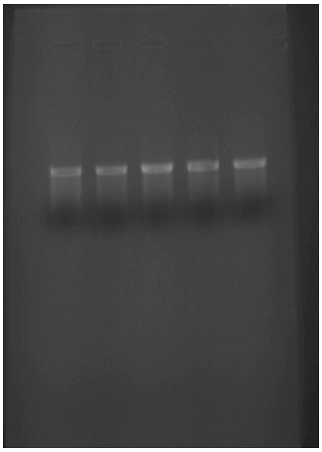

Raw Fig.3B

#B1-3

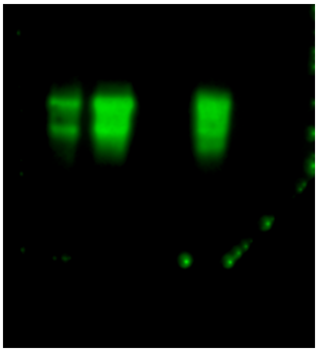

SYSY

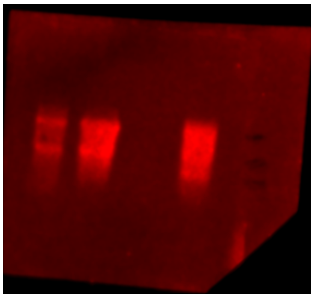

EtBr

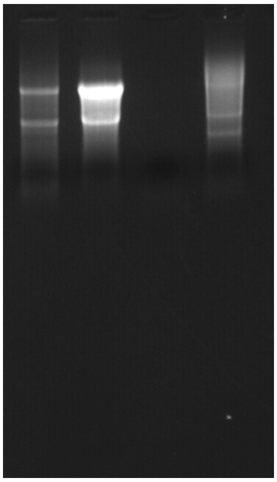

Raw Fig 3C

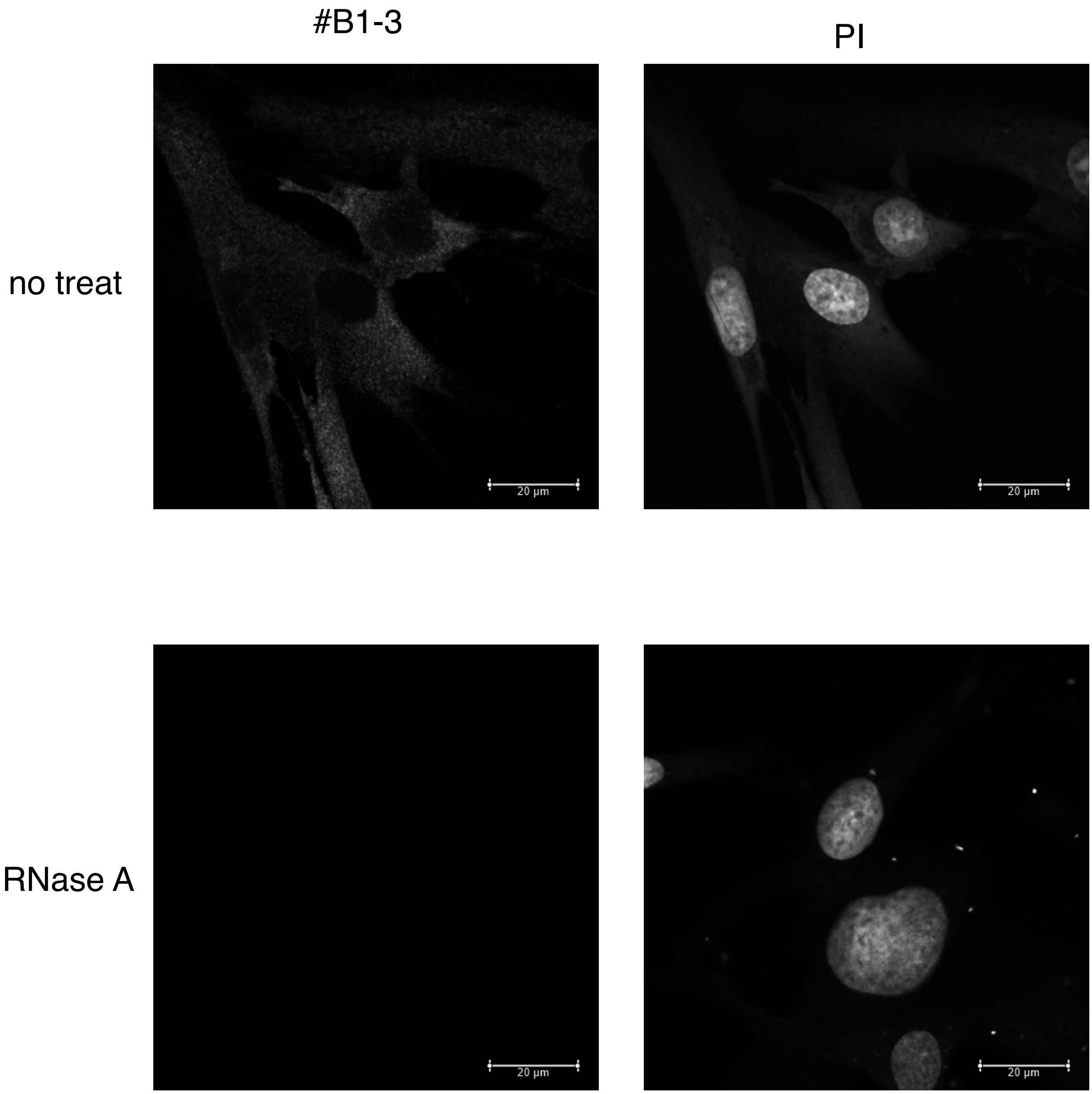

Raw Fig 3D

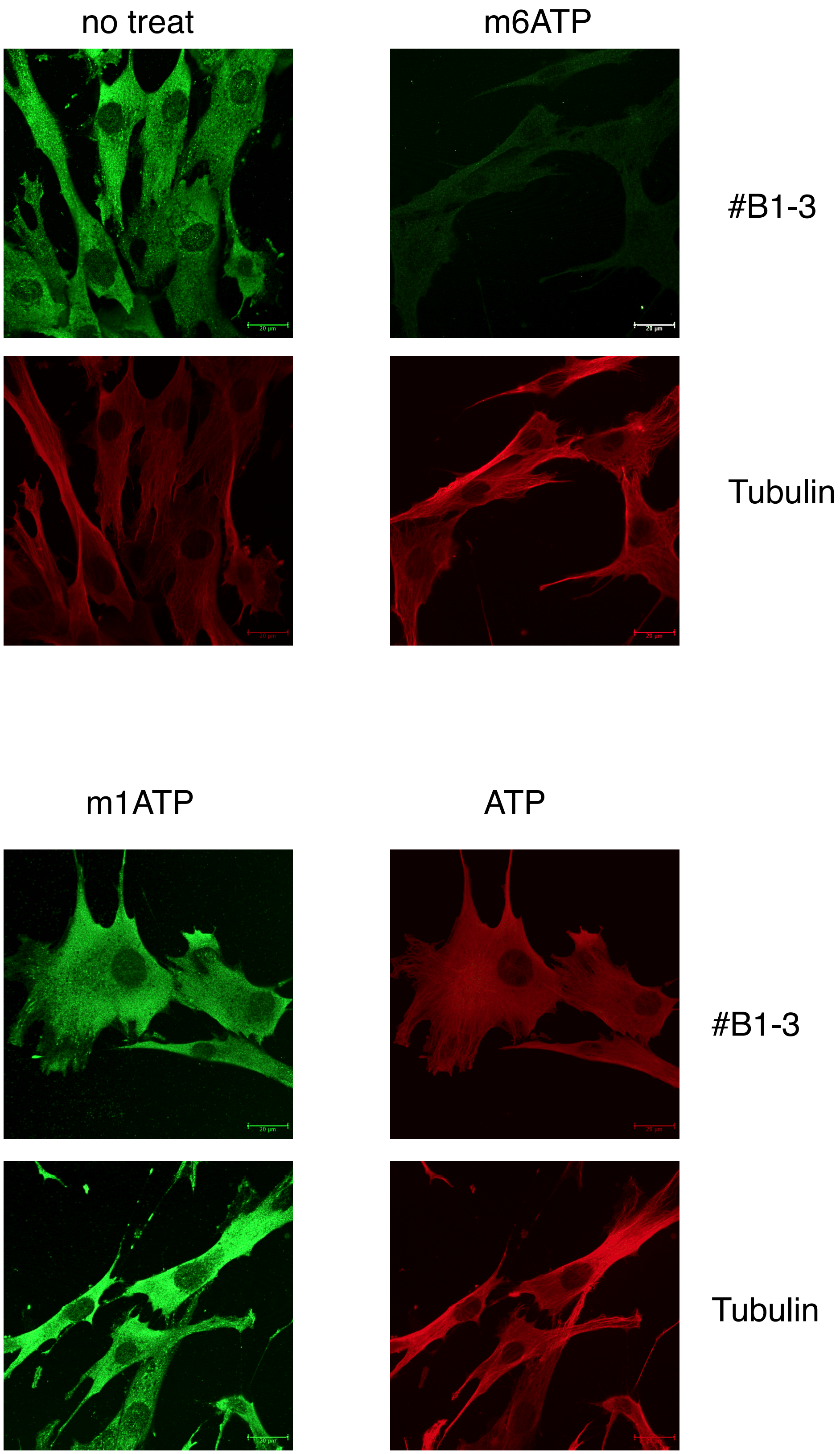

Raw Fig 4B

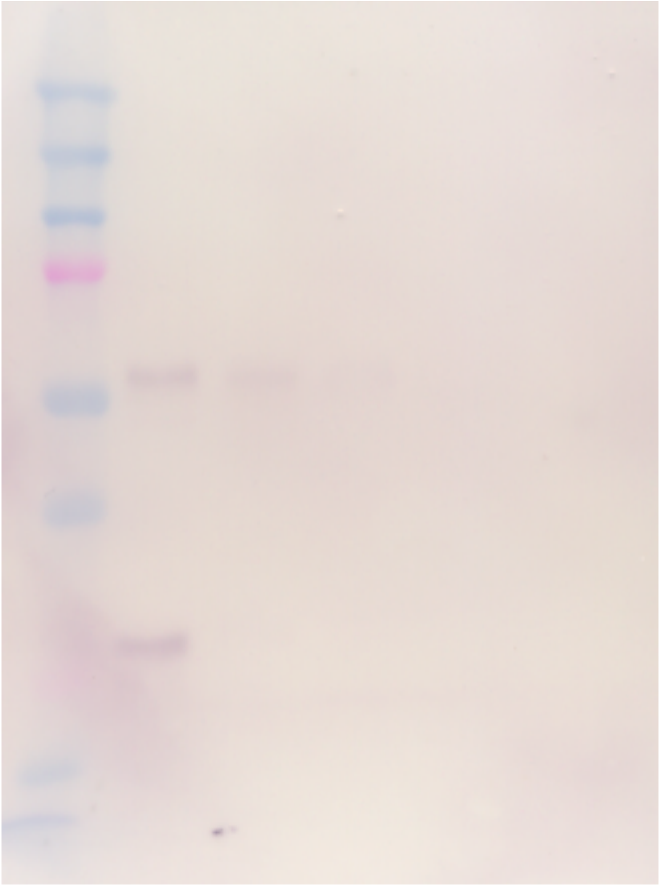

Raw S1 Fig B

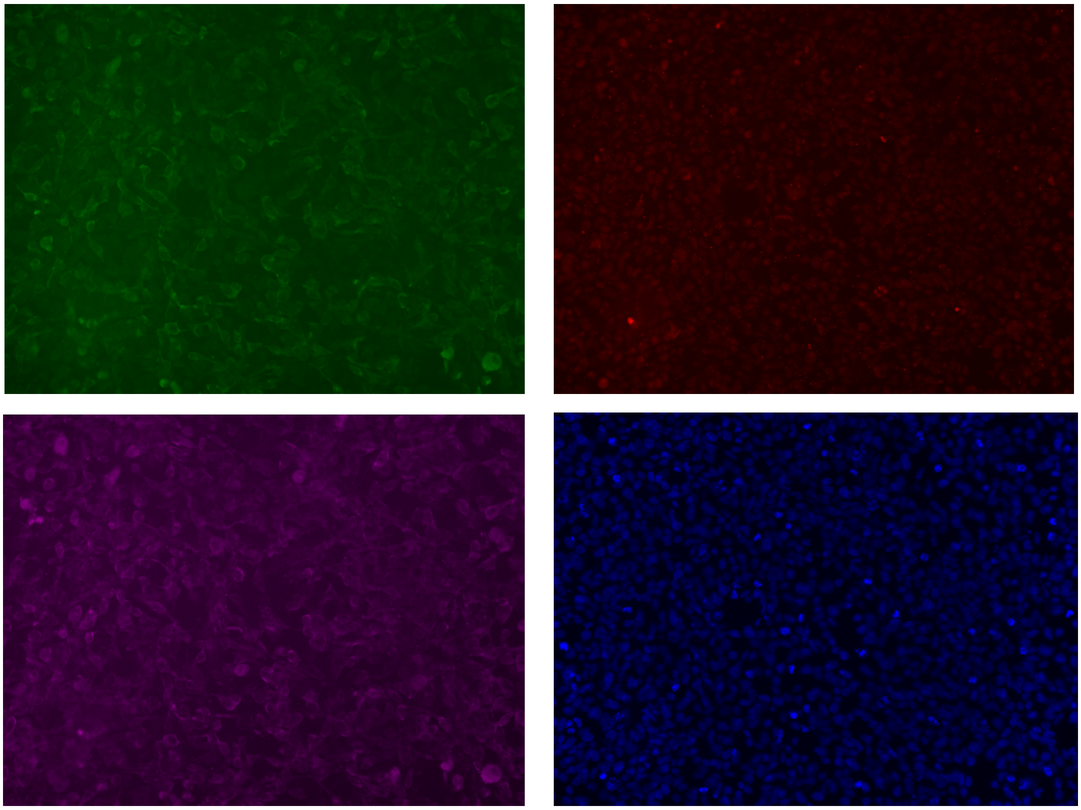

Raw S3 Fig B

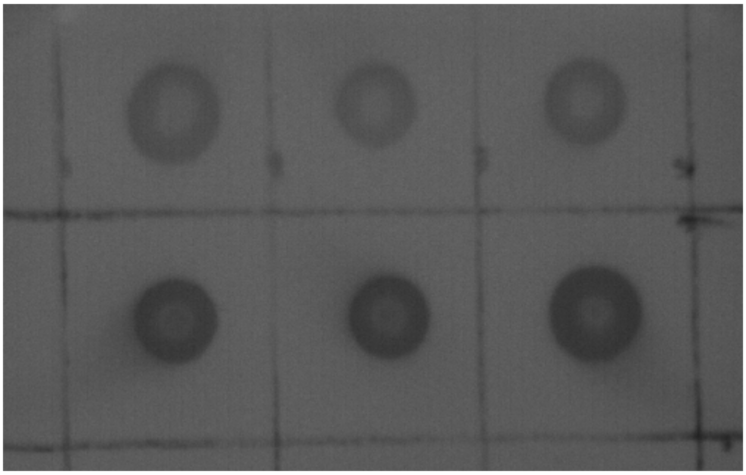

Raw S4 Fig A

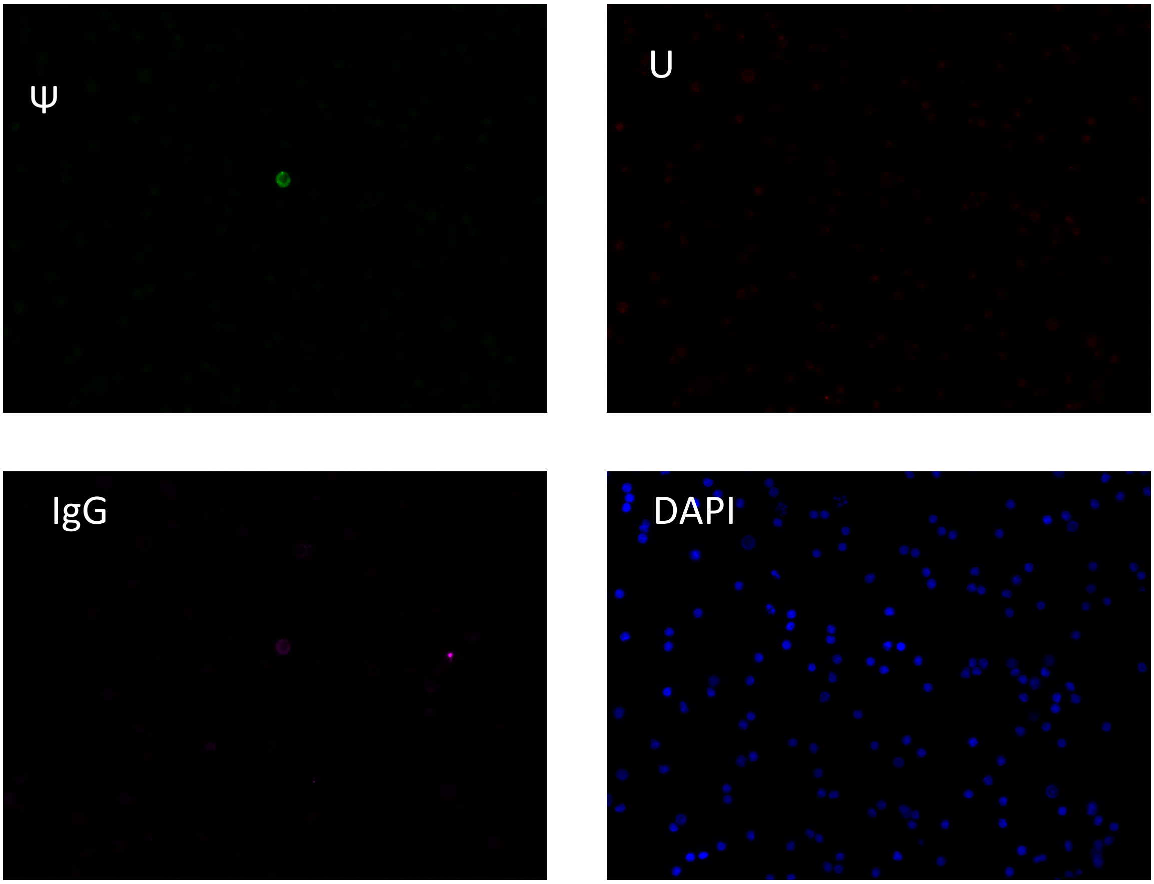

Raw S4 Fig C

#3-19

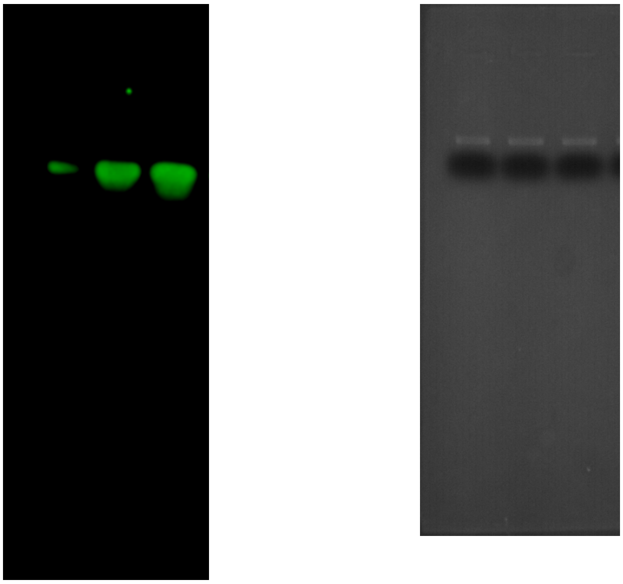

EtBr
